# InGaN Light-Emitting Diodes with an Embedded Nanoporous GaN Distributed Bragg Reflectors

**DOI:** 10.1038/srep29138

**Published:** 2016-07-01

**Authors:** Guo-Yi Shiu, Kuei-Ting Chen, Feng-Hsu Fan, Kun-Pin Huang, Wei-Ju Hsu, Jing-Jie Dai, Chun-Feng Lai, Chia-Feng Lin

**Affiliations:** 1 Department of Materials Science and Engineering, National Chung Hsing University, Taichung, 402, Taiwan; 2 Department of Photonics, Feng Chia University, 100, Wenhwa Road, Seatwen, Taichung 40724, Taiwan

## Abstract

InGaN light emitting diodes (LED) structure with an embedded 1/4λ-stack nanoporous-GaN/undoped-GaN distributed Bragg reflectors (DBR) structure have been demonstrated. Si-heavily doped GaN epitaxial layers (n^+^-GaN) in the 12-period n^+^-GaN/u-GaN stack structure are transformed into low refractive index nanoporous GaN structure through the doping-selective electrochemical wet etching process. The central wavelength of the nanoporous DBR structure was located at 442.3 nm with a 57 nm linewidth and a 97.1% peak reflectivity. The effective cavity length (6.0λ), the effective penetration depth (278 nm) in the nanoporous DBR structure, and InGaN active layer matching to Fabry-Pérot mode order 12 were observed in the far-field photoluminescence radiative spectra. High electroluminescence emission intensity and line-width narrowing effect were measured in the DBR-LED compared with the non-treated LED structure. Non-linear emission intensity and line-width reducing effect, from 11.8 nm to 0.73 nm, were observed by increasing the laser excited power. Resonant cavity effect was observed in the InGaN LED with bottom nanoporous-DBR and top GaN/air interface.

Gallium nitride (GaN) materials have considerable in optoelectronic devices such as light-emitting diodes (LEDs), laser diodes (LD)^1^, and vertical cavity surface emitting lasers (VCSEL)^2^. High reflectivity distributed Bragg reflectors (DBR) structure, short cavity thickness^3^,^4^,^5^, high transparence conductive layer, efficient transverse current spreading, small current confinement aperture, and resonant cavity controled in the nitride VCSEL need to be improved. Leonard *et al*. reported on violet nonpolar III-nitride VCSELs with a tunnel junction intracavity contact^6^ and an Al ion implanted aperture^7^. The epitaxial AlGaN/GaN stack^8^,^9^ and AlN/GaN stacks^10^,^11^ structures had been reported for the bottom epitaxial DBRs in GaN-based VCSEL devices. Large lattice mismatch and low refractive index different of the stack structures are the challenges for the epitaxial DBR structures with long epitaxial growth time. The AlInN/GaN DBR structure^12^,^13^ is lattice matched to GaN material, but the growth of AlInN layer remains a challenge in InGaN-based LED structures. To realize the high reflectivity with less pairs of stack structure, the air-gap/GaN DBR structures with large refractive index different had been fabricated through selectively anodized processe^14^,^15^, and thermal decomposition techniques^16^,^17^,^18^. But, the low mechanical strength and the tiny high reflective area remains a challenge for the photonic device fabrication. Plawsky *et al*.^19^. reported the nanoporous material for the photonics through the evaporation induced self-assembly process and oblique or glancing angle deposition. The resonant cavity effect of III-nitride thin-film flip-chip light-emitting diodes with anatase TiO_2_ microsphere array were reported^20^. Nanoporous GaN material has been reported as an effective low refractive index for the DBR structure applications^21^,^22^,^23^.

In this paper, InGaN LED structure with bottom nanoporous-GaN DBR structure has been fabricated. Twelve pairs of n^+^-GaN:Si/undoped-GaN epitaxial stack structure were grown for the following selective electrochemically (EC) etching process. The n^+^-GaN:Si epitaxial layer was transformed into the nanoporous GaN structure through the EC-etching process. The effective cavity thickness consists of the top InGaN LED structure (800 nm) and the effective penetration depth in the nanoporous DBR structure (278 nm) that calculated from the angle-dependent far field photoluminescence spectra. InGaN active layer matched to the Fabry-Pérot mode were designed and analyzed. During the optical pumping measurement, the non-linear emission intensity, and line-width narrowing phenomenon were observed that indicated the resonant cavity effect on the InGaN/NP-DBR structure. Optical and electrical properties of the InGaN LED structure with and without nanoporous DBR structure were analyzed in detail.

## Results

The LED epitaxial layer consisted of a 30-nm-thick GaN buffer layer grown at 530 °C, a 2.0-μm-thick unintentionally doped GaN layer (u-GaN, 1050 °C, 5 × 10^16^ cm^−3^), twelve pairs of n^+^-GaN:Si/u-GaN stack structure, a 40 nm-thick u-GaN layer (1050 °C), a 540 nm-thick n-GaN layer (1050 °C, 2 × 10^18^ cm^−3^), ten pairs of In_0.2_GaN/GaN (3 nm/9 nm) multiple-quantum wells (MQWs, 760 °C), and a 100 nm-thick p-GaN layer (950 °C, 1 × 10^18^ cm^−3^). The parallel wet etching channels with a 260 μm-spacing on the LED structure were defined through a laser scribing (LS) process. The laser scribing depth was 2.2 μm to reach the as-grown 12-period n^+^-GaN/u-GaN stack structure by using a 355 nm laser. After the lateral wet etching process, the Si-heavy doped n^+^-GaN:Si layer was transformed into a nanoporous GaN layer through the doping-selective electrochemically etching process in a 0.5 M nitride acid solution at positive 8 V external bias voltage^24^. After the EC-etching process, a high refractive index n-type GaN:Si layer was transformed into a low refractive index nanoporous GaN layer. The treated DBR structure consisted of a quarter-wavelength (1/4λ) nanoporous GaN layer and a quarter-wavelength (1/4λ) undoped-GaN layer acted a bottom light reflector at about 442 nm central wavelength (λ). Then, a 150-nm-thick indium tin oxide (ITO) film was deposited on the mesa region acted as a transparent conductive layer. The ITO layers on the p-type GaN:Mg layer was annealed in furnace at 600 °C for 20 min in order to improve the ohmic contact property. Then, the patterned Ti/Al (50 nm/200 nm) metal layers were deposited as the bottom n-GaN conductive layer for the n-type contact metal layer.

**Figure 1:**
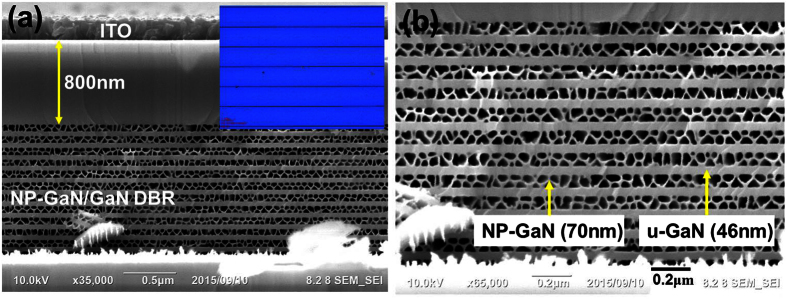
(**a**) The cross-sectional SEM micrograph of the DBR-LED structure was observed. The OM image of the DBR-LED is inserted with a blue light image. (**b**) 70 nm-thick NP-GaN layer and 46 nm-thick GaN layer were observed in the stacked DBR structure.

In Fig. 1(a), the 800 nm-thick InGaN LED structure and the bottom NP-GaN/u-GaN DBR structure were observed in the cross-sectional SEM micrograph. The ITO layer was deposited on the top of the p-GaN:Mg layer as an electrode contact layer for EL measurement. The OM image of the EC treated DBR-LED with the laser scribing lines and the bottom nanoporous DBR structure is inserted in Fig. 1(a). The etching process occurs from the laser scribing channels when the nitride etching solution comes in contact with the n^+^-GaN layers in the DBR stack structure. Under a positive bias voltage condition, the biased carriers are located at the low resistivity n^+^-GaN:Si layer to attract the etching etchant for the wet etching process. After the wet etching process, the uniformly blue reflected light can be observed on the EC-etched structures as shown in Fig. 1(a) indicating that the n^+^-GaN layers in the stack structure have been transformed into the nanoporous structure. The NP-DBR stacked structure consisted of a 70 nm-thick NP-GaN layer and 46 nm-thick GaN layer as shown in Fig. 1(b). The NP-GaN/u-GaN etching interface was observed clearly due the high doping-selectively EC etching process. The doping-selectivity EC etching process occurred on the n^+^-GaN:Si layers in the n^+^-GaN:Si/u-GaN stack structure. The n^+^-GaN:Si layers were etched as the nanoporous GaN structure that provided the etching channel for the nitride acid solution in the later etching process. Under a positive bias voltage condition, the biased carriers were located at the low resistivity n^+^-GaN:Si layer to attract the etching etchant through the wet-etched nanoporous channels. During the EC etching process, the n^+^-GaN:Si layer was oxidized and the oxide was dissolved subsequently into the acid solution where the nanoporous channel was formed^25^.

**Figure 2:**
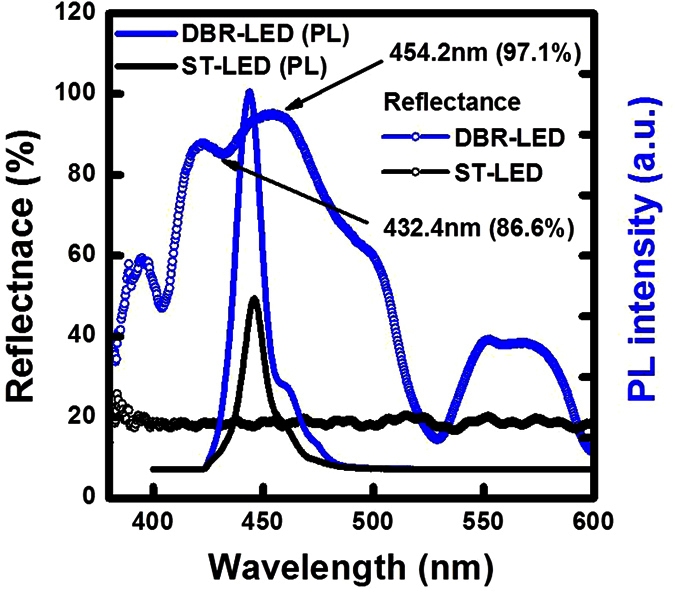
Reflectance spectra and PL emission spectra were measured in InGaN-LED with and without nanoporous DBR structure.

In Fig. 2, the reflectance spectra were measured on the ST-LED and the DBR-LED structure by using the Xe lamp as reference light source. The reflectivity of ST-LED is measured at about 18% with interference fringes due to Fabry-Pérot (FP) effect on the flat LED epitaxial layer. After the EC-etching process, high light reflectivity was observed in the DBR-LED structure. The central wavelength of the reflectance spectrum was located at 442.3 nm with a 57 nm linewidth that was measured on the DBR-LED structure with top InGaN cavity structure and bottom NP-DBR structure. The wavelength and the reflectivity of the DBR-LED structure were measured at 454.2 nm/97.1% for the peak reflectance and 432.4 nm/86.6% for the dip position, respectively. The dip signal of the reflectance spectrum is caused by the FP effect in the top InGaN LED structure with top GaN/air interface and bottom GaN/NP-DBR. The twelve-period stack structure consisted of 1/4λ nanoporous GaN layer and 1/4λ u-GaN layer at 442 nm. The peak wavelengths and line-width of the PL spectra were measured at 446.3 nm/16.2 nm for the ST-LED and at 443.8 nm/15.9 nm for the DBR-LED structure, respectively. The PL emission wavelength of the DBR-LED is almost matched to the central wavelength of the DBR reflector that enhanced the PL emission intensity.

**Figure 3:**
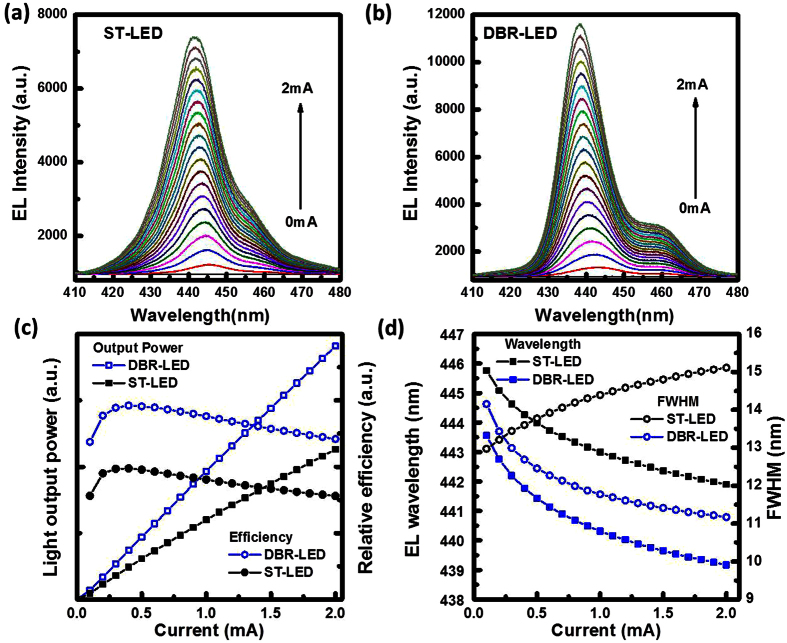
The EL emission spectra of (**a**) ST-LED and (**b**) DBR-LED were measured by varying the injection current. (**c**) The light output power and the external quantum efficiency of both LED structures were measured. (**d**) The emission wavelength and the line-width of both LED structures were observed. The line-width narrowing effect was observed in DBR-LED.

The EL spectra of the both LED structures were measured by varying the injection current from 0.1 to 2.0 mA, as shown in Fig. 3(a,b). The EL emission intensity of the DBR-LED was stronger than it of the ST-LED structure. The EL emission spectra of the DBR-LED structure were affected by the reflectance spectrum of the bottom nanoporous DBR structure. In Fig. 3(c), the light output power and the external quantum efficiency of both LED structures were measured by varying the injection current. The relative output power of the DBR-LED had a 68% enhancement compared with the ST-LED structure at 2 mA (41 A/cm^2^ current density). The peak efficiency of both LED structures was measured at 0.4 mA operation current. The external quantum efficiency were slight decreased by increasing the injection current that the efficiency droop were calculated at 21% for ST-LED and at 17% for DBR-LED, respectively. The EL emission wavelengths and the linewidth as a function of injection current were shown in Fig. 3(d). The EL emission wavelength were measured at 441.9 nm for the ST-LED and 439.2 nm for the DBR-LED, respectively, at 2 mA. The EL emission wavelength of the DBR-LED structure was slightly blueshifted compared to the ST-LED structure that indicated the compressive strain in InGaN active layer was partially released by forming the bottom nanoporous DBR structure. The compressive strain induced piezoelectric effect in InGaN active layer was caused by the lattice mismatch at the GaN/sapphire and GaN/InGaN interfaces. In the ST-LED structure, the linewidths of the EL spectra were slightly broadened from 13.0 nm (0.1 mA) to 15.1 nm (2 mA). In the DBR-LED structure, the EL linewidths were reduced from 14.2 nm (0.1 mA) to 11.2 nm (2 mA) that caused by the resonant cavity effect. The EL emission peak wavelength was close to the resonant dip in the reflectance spectra as shown in Fig. 2. In the EL emission properties, the embedded nanoporous DBR structure acted a reflector and strain released layer to improve the external quantum efficiency in DBR-LED structure. The line-width narrowing effect of the EL spectra was observed in the DBR-LED structure that indicated the resonant cavity effect between top GaN/air and bottom DBR reflectors.

**Figure 4:**
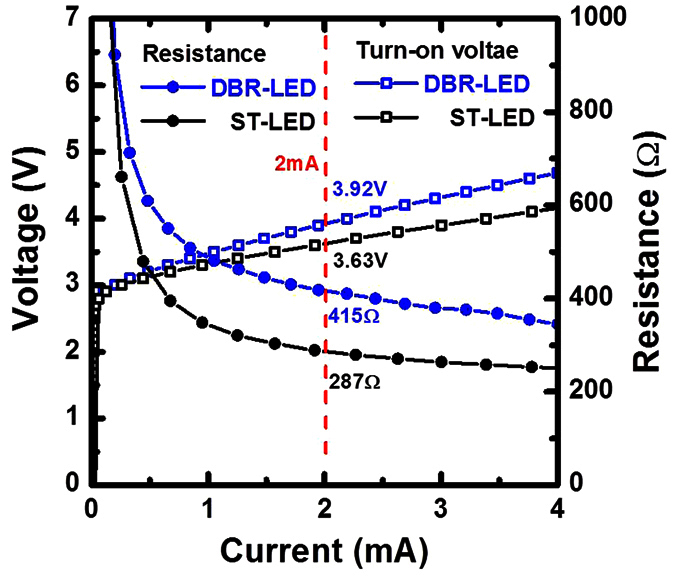
The operational voltage and the series resistance of ST-LED and DBR-LED were measured by varying the operating current.

The turn on voltage of both LED structures was about 2.9 V. At 2 mA operation current, the operation voltage and the resistance were observed at 3.63 V/287 Ω for the ST-LED and at 3.92 V/415 Ω for the DBR-LED, respectively, as shown in Fig. 4. By forming the nanoporous GaN DBR structure, the effective thickness of the LED structure was reduced to 800 nm above the nanoporous DBR structure so that the resistivity of the DBR-LED would be slightly increased. The u-GaN layers in the stack structure are unintentionally doped GaN layers caused by the epitaxial growth process where the carrier concentration is about 5 × 10^16^ cm^−3^. With electrode assisted etching with positive biasing, the u-GaN layers are the electrical conductivity layers that come in contact with the n^+^-GaN:Si layers in the stack structure. Therefore, the n^+^-GaN:Si layers can be totally etched as the nanoporous GaN layers. By forming the nanoporous GaN DBR structure, the effective thickness of the LED structure is reduced to 800 nm above the nanoporous DBR structure where the resistivity of the DBR-LED is slightly increased.

**Figure 5:**
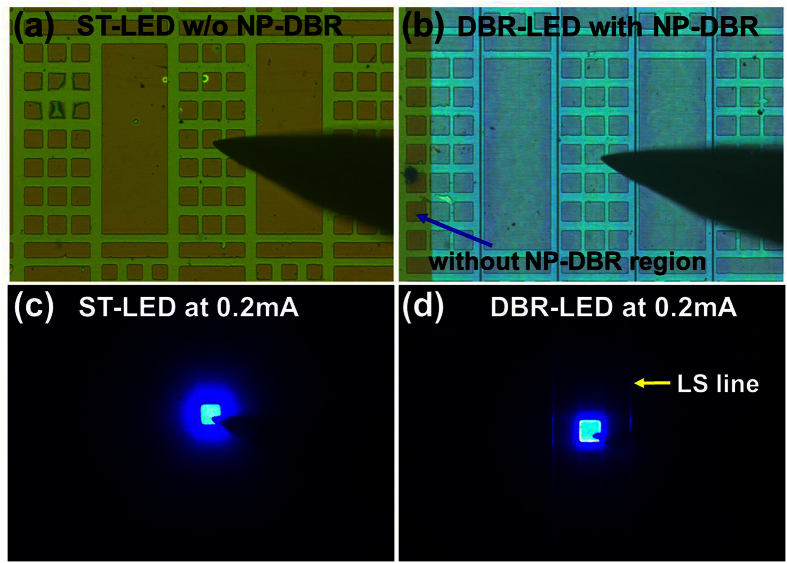
The OM images of the (**a**) ST-LED and (**b**) DBR-LED with the 70 × 70 μm^2^ ITO patterns were observed for EL measurement. The interface of the LED with and without NP-DBR is observed. The EL emission images of (**c**) ST-LED and (**d**) the DBR-LED were measured at 0.2 mA operating current.

The OM images of the ST-LED and the DBR-LED with the 70 × 70 μm^2^ ITO patterns were observed in Fig. 5(a,b), respectively. Both the ST-LED and DBR-LED were located at the same sample for comparing the optical properties. In Fig. 5(b), the interface of the LED with and without bottom NP-DBR structure is observed. The lateral etching width on the NP-DBR structure is about 130 μm from the laser scribing line which has some non-treated region for fabricating the ST-LED devices. EL emission images of the ST-LED and the DBR-LED were measured at 0.2 mA operating current as shown in Fig. 5(c,d), respectively. In the ST-LED, the EL emission intensity is uniformly distributed on the ITO pattern region as shown in Fig. 5(c). The EL emission light is propagated outwardly surrounding the ITO pattern due to the light guide effect in the air/GaN/sapphire structure. In the DBR-LED, the EL emission intensity is localized at the ITO pattern region as shown in Fig. 5(d). The light propagated length is shorter than the ST-LED structure because the EL emission light can be reflected to the normal direction by the bottom NP-DBR structure. The embedded nanoporous DBR layer is close to the InGaN active layer to improve the light reflection and the light extraction at the normal direction of the LED wafer.

**Figure 6:**
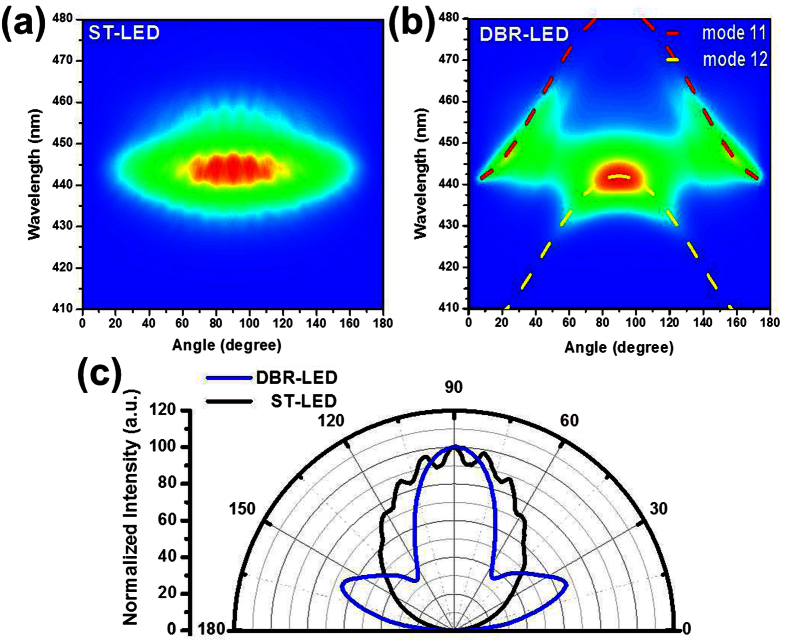
The PL emission spectra of (**a**) the ST-LED and (**b**) the DBR-LED were measured through the angle-resolved PL measurements using a 405 nm diode laser as an excitation laser source. (**c**) The normalized far-field radiation pattern of both LED structures were observed.

In Fig. 6, the PL spectra were analyzed through angle-resolved photoluminescence measurements with a 405 nm excitation laser illuminated from the backside sapphire substrate. From the backside laser illumination, the InGaN active layer had been excited by the 405 nm laser. The laser light is not absorbed by the sapphire substrate, the n-type GaN:Si layer, and the p-type GaN:Mg layer. But in the DBR-LED structure, the 405 nm laser power will be partially reflected by the bottom nanoporous GaN DBR reflector. The PL emission spectra were measured by a multi-channel CCD detector with a 550 mm focal length monochromator. The PL spectra was detected at the front-side of the flat LED wafer without chip process. In Fig. 6(a), the Fabry–Pérot (FP) interference line-patterns as a function of the detected angle were observed in the ST-LED structure grown on the patterned sapphire substrate. The interferences of the EL spectra were observed in the ST-LED structure that has the flat top GaN:Mg/Air interface and the bottom GaN/patterened-sapphire interface. The FP line pattern is not so clear because of the partial light scattering at the GaN/patterned-sapphire interface. The thicknesses of the epitaxial layers were calculated at 4.2 μm for the ST-LED which was similar to the actual thicknesses measured by the cross-section SEM images of the non-treated epitaxial structures. In the DBR-LED structure, the two lines pattern was observed in far-field PL spectra were caused by the top GaN:Mg/Air interface and the bottom nanoporous DBR reflector as shown in the Fig. 6(b). The angular PL spectra of the InGaN active layer were measured in the DBR-LED structure. The intensity formula of the angular dependent spectra is I(λ_o_,θ) = (2n_s_T/mc)cos(θ), where n_s_ is the refractive index of GaN (2.461), m_c_ is the cavity mode number (integer), and T is the cavity thickness^26^. From the calculated results, the effective cavity thickness is about 1078 nm. The FP mode number from 11 (red dash line) to 12 (yellow dash line) were observed above the air cone as shown in Fig. 6(b). The thickness of the InGaN LED structure was measured at about 800 nm above the nanoporous DBR structure. The FP mode order 12 in the 6.0λ resonant cavity is matched to the PL emission wavelength of the InGaN active layer. From the simulation results, the penetration depth in the nanoporous DBR structure is about 278 nm (about 1.5λ) including in the effective resonant cavity^27^. The treated DBR-LED structure consisted of an 800 nm-thick (about 4.5λ) LED layer and a bottom NP-DBR structure measured from the cross-sectional SEM image in Fig. 1(a). The normalization PL far-field radiation patterns of both LED structures were observed in Fig. 6(c). By forming the embedded NP-DBR structure, the divergent angle of the DBR-LED was narrowing at 52^o^ compared with the ST-LED structure at 98^o^. Peak emission intensities of the DBR-LED are observed at 23^o^, 90^o^ (normal direction), and 157^o^.

**Figure 7:**
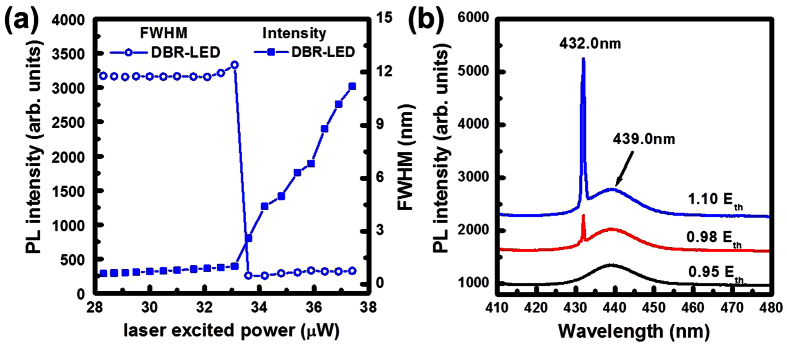
(**a**) The PL intensities and the linewidth of the optical pumping spectra were measured as a function of the laser excitation power. (**b**) The emission spectra of the DBR-LED structure were observed under 0.95E_th_, 0.98E_th_, and 1.10E_th_ laser pumping power.

Optical pumping spectra of the DBR-LED structure were measured as shown in Fig. 7 by using an Nd-YVO_4_ 355 nm pulse laser as an excitation laser source. In Fig. 7(a), the PL intensities and the linewidth of the optical pumping spectra were measured as a function of the laser excitation power. By increasing the laser excited power, the PL emission intensities had a non-linear increasing property and the line-width has the narrowing phenomenon. The threshold laser pumping power (E_th_) was observed at 33.1 μW. In Fig. 7(b), the emission spectra of the DBR-LED structure were observed under 0.95E_th_, 0.98E_th_, and 1.10E_th_ laser pumping power, respectively. The line-width of the InGaN emission spectra was reduced from 11.80 nm for the typical InGaN active layer (at 439.0 nm) to 0.73 nm for the stimulation emission peak (at 432.0 nm). The intensity of stimulated emission peak at 439.0 nm become strongly and narrowed down to 0.73 nm by increasing the laser pumping power. The reduction in the FWHM of the optical pumping spectra was caused by the resonance cavity effect between the top air/GaN:Mg interface (with about 18% reflectance) and the bottom GaN/nanoporous-DBR reflectors (with about 97% reflectance). At a low laser excitation power, low emission intensity and a broaden line-width (FWHM) of the PL spectra are caused by the spontaneous emission from the InGaN active layer. When the laser excited power is higher than the threshold laser pumping power (E_th_), the emission line-width has a narrowing phenomenon that indicates a stimulated emission in InGaN resonance cavity. This indicated the high reflectance nanoporous DBR structure with the InGaN cavity layer is demonstrated in the DBR-LED structure.

## Discussion

An InGaN LED structure with an embedded nanoporous DBR structure has been demonstrated. N^+^-GaN:Si layers of a twelve n^+^-GaN:Si/u-GaN pairs structure were transformed into nanoporous GaN layers with a low refractive index property through a selective electrochemical etching process. High reflectivity (>97%) and a wide treated area (260 μm-width) of a nanoporous DBR structure were fabricated on normal-sized LED chips that enhanced the light output power. High reflectivity in the NP-DBR was demonstrated which matched to the PL emission wavelength and the cavity length of the InGaN active layer. The effective cavity length, the penetration depth in NP-DBR structure, the InGaN active layer matched to a Fabry-Pérot mode, and the narrowing divergent angle were analyzed from the far-field PL radiative spectra. High EL emission intensity and a line-width narrowing effect were measured in the DBR-LED and compared to the ST-LED structure. When compared with the traditional AlGaN/GaN and AlN/GaN epitaxial DBR structures with the long epitaxial growth times, the treated n^+^-GaN:Si/u-GaN stacks structure is comparable to the conventional epitaxial growth process in the commercialized InGaN LED structure. During the optical pumping measurement, the non-linear emission intensity and the line-width narrowing phenomenon, from 11.8 nm to 0.73 nm, were observed, indicating a resonant cavity effect on the InGaN/NP-DBR structure. The resonant cavity effect observed in the InGaN-LED/NP-DBR structure has the potential for a high efficiency InGaN-based optoelectronic device and for VCSEL applications.

## Methods

### Epitaxial growth

InGaN LED structures were grown on a 2 in. optical-grade c-face (0001) pattern sapphire substrate using a metal organic chemical vapor deposition system. Trimethylgallium (TMGa), trimethylindium (TMIn), and ammonia (NH_3_) were used as gallium (Ga), indium (In), and nitrogen (N) sources material, respectively. Silane (SiH_4_) and biscyclopentadienyl magnesium (CP_2_Mg) were used as the n-type doping and p-type doping sources, respectively.

### Optical characterization

The geometric morphologies of the LED structures were observed using polarized optical microscopy (OM) and a field-emission scanning electron microscope (FE-SEM, JEOL 6700F). The light intensity profiles of the LED chips were measured using a beam profiler (Spiricon: number of effective pixels: 1600 × 1200 pixels). Light output power and far-field radiation patterns were measured on non-encapsulated LEDs in chip form. The photoluminescence (PL) spectra of both LED structures were obtained by an angle-resolved PL measurement by using a 405 nm diode laser as an excitation laser source. The optical properties of the LED samples were measured through angle-resolved electroluminescence (EL) measurement by using monochromators (JOBIN YVON iHR550) with a TE-cooled charge-coupled device (CCD) detector.

## Additional Information

**How to cite this article**: Shiu, G.-Y. *et al*. InGaN Light-Emitting Diodes with an Embedded Nanoporous GaN Distributed Bragg Reflectors. *Sci. Rep*. **6**, 29138; doi: 10.1038/srep29138 (2016).
